# Stretch-Induced Down-Regulation of HCN2 Suppresses Contractile Activity

**DOI:** 10.3390/molecules28114359

**Published:** 2023-05-26

**Authors:** Job Baffin Kola, Botagoz Turarova, Dora Csige, Ádám Sipos, Luca Varga, Bence Gergely, Farah Al Refai, Iván P. Uray, Tibor Docsa, Karen Uray

**Affiliations:** 1 Department of Medical Chemistry, School of Medicine, University of Debrecen, 4032 Debrecen, Hungary; baffin.kola@med.unideb.hu (J.B.K.); botagoz.turarova@nu.edu.kz (B.T.); csige.dora@med.unideb.hu (D.C.); sipos.adam@med.unideb.hu (Á.S.); varga.luca@med.unideb.hu (L.V.); gergely.bence@med.unideb.hu (B.G.); farahalrefai66@gmail.com (F.A.R.); tdocsa@med.unideb.hu (T.D.); 2 Department of Clinical Oncology, Faculty of Medicine, University of Debrecen, 4032 Debrecen, Hungary; uray.ivan@med.unideb.hu

**Keywords:** intestinal motility, hyperpolarization-activated and cyclic nucleotide-gated 2 channel, mechanotransduction, ileus

## Abstract

Although hyperpolarization-activated and cyclic nucleotide-gated 2 channels (HCN2) are expressed in multiple cell types in the gut, the role of HCN2 in intestinal motility is poorly understood. HCN2 is down-regulated in intestinal smooth muscle in a rodent model of ileus. Thus, the purpose of this study was to determine the effects of HCN inhibition on intestinal motility. HCN inhibition with ZD7288 or zatebradine significantly suppressed both spontaneous and agonist-induced contractile activity in the small intestine in a dose-dependent and tetrodotoxin-independent manner. HCN inhibition significantly suppressed intestinal tone but not contractile amplitude. The calcium sensitivity of contractile activity was significantly suppressed by HCN inhibition. Inflammatory mediators did not affect the suppression of intestinal contractile activity by HCN inhibition but increased stretch of the intestinal tissue partially attenuated the effects of HCN inhibition on agonist-induced intestinal contractile activity. HCN2 protein and mRNA levels in intestinal smooth muscle tissue were significantly down-regulated by increased mechanical stretch compared to unstretched tissue. Increased cyclical stretch down-regulated HCN2 protein and mRNA levels in primary human intestinal smooth muscle cells and macrophages. Overall, our results suggest that decreased HCN2 expression induced by mechanical signals, such as intestinal wall distension or edema development, may contribute to the development of ileus.

## 1. Introduction

Feeding intolerance and the development of ileus are common complications in trauma, surgery, and critically ill patients [1,2,3]. Virtually all abdominal surgery patients experience some degree of ileus, and 20–90% of other surgical patients develop postoperative ileus [1]. Furthermore, gastrointestinal complications are the leading cause of hospital readmissions after non-cardiac surgeries [4]. Approximately 33% of moderate-to-severe trauma patients develop feeding intolerance, and 25% of these trauma patients have documented ileus [2]. Despite numerous strategies to treat ileus, the incidence of ileus has increased in the last few decades [5]. Ileus significantly increases patient care costs by necessitating the treatment of consequent complications, feeding parenteral nutrition, hospital readmissions, and prolonging hospital and intensive care unit stays [6,7]. In addition, the development of ileus causes significant patient discomfort and malnutrition and increases the risk of septic, pulmonary, and thromboembolic complications [8,9]. Thus, ileus is a significant clinical problem and economic burden. Understanding how ileus develops is important in developing new treatment strategies.

Both experimental and clinical data demonstrate that inflammatory insult plays a critical role in the development of ileus [2,10,11,12,13,14]; however, the contribution of mechanotransduction has not been widely explored. As ileus develops, the intestines become distended, exerting mechanical force on the smooth muscle layers. We previously demonstrated that increased stretch of intestinal smooth muscle suppresses contractile activity via the down-regulation of MYPT1 and myosin light chain phosphorylation [15,16]. Furthermore, systemic inflammation develops in most trauma or critically ill patients, causing intestinal edema. Intestinal edema develops after intestinal manipulation, as would occur during exploratory laparotomy, and may also develop during trauma resuscitation [11,15,17]. Intestinal edema increases tension of the gut wall and alters gene expression [18,19,20]. Thus, changes in mechanical forces in the gut wall for even a relatively short period (4–12 h) can cause dysregulation of the contractile apparatus, including both protein and gene expression changes [11,15,16,17,18,19,21]. However, the mechanisms by which mechanical stretch affects downstream signaling pathways in intestinal smooth muscle are unclear. To better understand the effects of mechanotransduction on signaling pathways in the small intestine, changes in gene expression were examined in intestinal smooth muscle after the induction of ileus compared to sham-operated controls [19]. Ileus was induced in this model by inducing intestinal edema, which causes increased stretch in the intestinal smooth muscle [17,20]. Expression changes in mechanosensitive ion channels were examined, and the down-regulation of hyperpolarization-activated and cyclic nucleotide-gated 2 channel (HCN2) during the development of intestinal edema and subsequent ileus was discovered (microarray data can be found at (http://www.ncbi.nlm.nih.gov/geo/query/acc.cgi?acc_GSE13193; accessed on 1 April 2023) [19].

HCN channels were originally identified in cardiac pacemaker cells, but have subsequently been detected in a wide variety of tissues, including the gut and smooth muscles of other organs [22,23,24,25,26,27,28,29]. The HCN family includes four isoforms (HCN1–4). The six-transmembrane subunits form homo- or heterotetramers to create a channel [30]. These unique channels conduct Na^+^ and K^+^, are activated by membrane hyperpolarization, and are modulated by cyclic nucleotides [30]. Loss of HCN2 causes malnutrition, leading to severe growth restriction [31,32]. In addition, HCN2 is highly expressed in myenteric cholinergic neurons, suggesting that HCN2 plays an important role in gastrointestinal motility [33]. We found that HCN2 is down-regulated in intestinal smooth muscle in an ileus model [19]. The purpose of this study was to determine the effects of HCN inhibition on intestinal motility.

## 2. Results

### 2.1. Effects of HCN Inhibition on Spontaneous Contractile Activity

**Figure 1:**
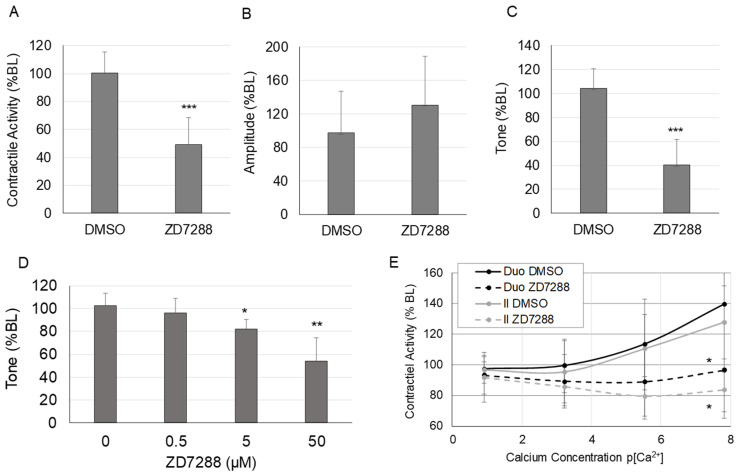
Changes in spontaneous contractile activity in response to HCN inhibition. (**A**) Spontaneous contractile activity (integral), (**B**) average contraction amplitude, and (**C**) tone (average minimum of the contractile cycle) in the ileum in response to ZD7288 (50 μM), (**D**) dose–response for the effects of HCN inhibition with ZD7288 on intestinal tone, (**E**) changes in calcium sensitivity in the ileum and duodenum in response to HCN inhibition with ZD7288. (*n* = 6 per group for Panels (**A**–**C**,**E**); *n* = 5 per group for Panel (**D**); Panels (**A**–**D**), *, *p* < 0.05 vs. DMSO only; **, *p* < 0.005 vs. DMSO only; ***, *p* < 0.0005 vs. DMSO only; Panel (**E**), *, *p* < 0.05 vs. DMSO at the same calcium concentration).

The changes in spontaneous contractile activity in response to HCN inhibition with ZD7288 are shown in Figure 1. Spontaneous intestinal contractile activity, calculated as the integral from 0, decreased significantly after 5 min of HCN inhibition with ZD7288 (50 μM) (*p* < 0.001; Figure 1A). (Contractile activity did not decrease significantly from minutes 1–5 of HCN inhibition.) Contraction amplitude did not change significantly after HCN inhibition (*p* < 0.12; Figure 1B); however, tone, measured as the average minimum of the contraction cycle, decreased significantly in response to HCN inhibition (*p* < 0.001; Figure 1C). The effects of ZD7288 on tone were dose-dependent (Figure 1D). The significant effects of ZD7288 on intestinal contractile activity were confirmed in a separate experiment. To determine if the effects of HCN inhibition were on intestinal smooth muscle, calcium sensitivity was measured in the duodenum and ileum in the presence or absence of ZD7288 (Figure 1E). HCN inhibition significantly suppressed the calcium sensitivity of spontaneous contractile activity compared to the vehicle control in both the duodenum and ileum (*p* < 0.05 at the highest calcium concentration).

### 2.2. Effects of HCN Inhibition on Agonist-Induced Contractile Activity

**Figure 2:**
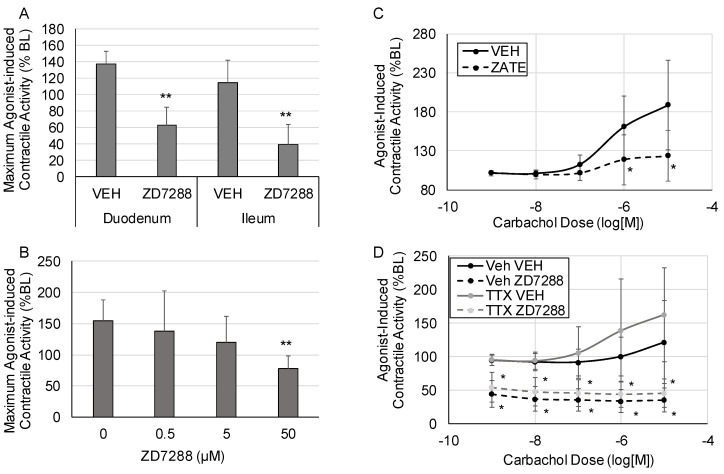
Changes in agonist-induced contractile activity. Tissue sections were treated with escalating doses of carbachol at 5 min intervals, and contractile activity was measured at each dose. (**A**) Maximum agonist-induced contractile activity in the duodenum and ileum after treatment with vehicle (DMSO) or ZD7288 to inhibit HCN2. (**B**) Dose–response for the effects of HCN inhibition with ZD7288 on maximum contractile activity. (**C**) The effects of HCN inhibition with zatebradine on agonist-induced intestinal contractile activity. (**D**) The effects of HCN inhibition with ZD7288 on agonist-induced intestinal contractile activity in the presence or absence of tetrodotoxin (TTX). (VEH is DMSO only; *n* = 6 per group for Panel (**A**), *n* = 5 for Panels (**B**,**D**), *n* = 8 per group for Panel (**C**); Panels (**A**,**B**): *, *p* < 0.05 vs. VEH, **, *p* < 0.005 vs. VEH; Panels (**C**,**D**): *, *p* < 0.05 vs. the respective vehicle at the same carbachol dose).

Changes in agonist-induced contractile activity in response to HCN inhibition are shown in Figure 2. HCN inhibition significantly suppressed maximum contractile activity in response to 10^−5^ carbachol in both the ileum and the duodenum (Figure 2A) (*p* < 0.005 vs. vehicle for both the duodenum and ileum). HCN inhibition with ZD7288 induced a dose-dependent decrease in the maximum carbachol-induced contractile activity in the ileum (Figure 2B) (*p* < 0.005 vs. vehicle at the 50 μM dose). To confirm that the effects were due to HCN inhibition and not off-target effects, the effects of a second HCN inhibitor, zatebradine, were tested. Zatebradine also significantly suppressed the contractile response to carbachol in the ileum, as shown in the carbachol dose–response curve, after pretreatment with zatebradine (*p* < 0.05) (Figure 2C). To test if the effects of HCN inhibition on contractile activity were mediated via the smooth muscle or enteric neurons, we pretreated the tissue sections with tetrodotoxin (TTX, 1 μM), as shown in Figure 2D. TTX had no significant effects on agonist-induced intestinal contractile activity. Pretreatment with TTX did not block the suppression of agonist-induced contractile activity induced by HCN inhibition (50 μM ZD7288) (*p* < 05 vs. VEH in the presence or absence of TTX pretreatment).

### 2.3. Impact of Inflammation on the Effects of HCN Inhibition

**Figure 3:**
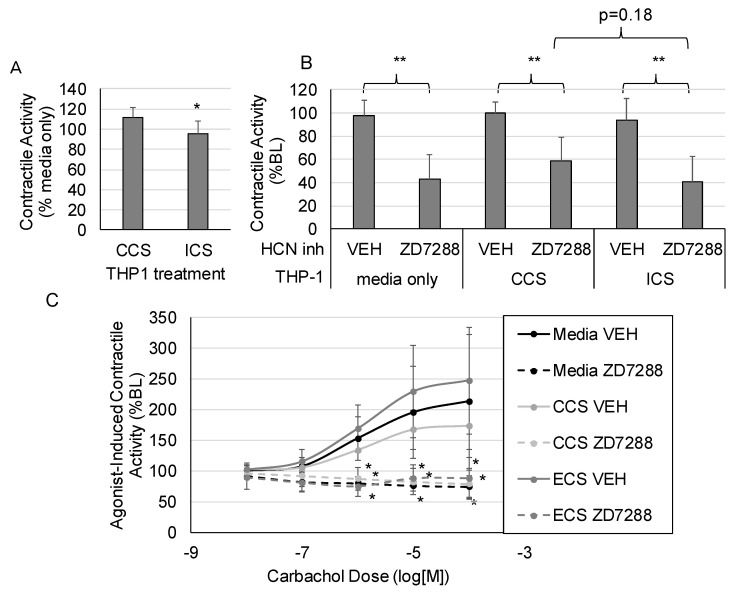
The impact of inflammation on HCN2 inhibition of intestinal contractile activity. THP1 cells were subjected to control or increased cyclical stretch for 4 h (see Methods for description of stretching protocols) and the “Condition media” were collected. Intestinal sections were treated with the media by replacing 10% of the organ chamber volume with the conditioned media. (**A**) The effects of conditioned media alone on spontaneous contractile activity (integral). (**B**) Changes in the inhibitory effects of HCN inhibition on spontaneous contractile activity in response to conditioned media. (**C**) Changes in the inhibitory effects of HCN inhibition on agonist-induced contractile activity in response to conditioned media. (VEH is DMSO only; *n* = 6 per group; Panel (**A**): *, *p* < 0.05 vs. CCS; Panel (**B**): *, *p* < 0.05 vs. VEH; **, *p* < 0.005 vs. VEH; Panel (**C**): *, *p* < 0.05 vs. the respective vehicle group for the same carbachol dose).

The impact of inflammation on HCN inhibition of intestinal contractility was investigated. Media were collected after subjecting stimulated THP1 cells (a monocytic cell line) to control cyclical stretch (CCS) or increased cyclical stretch (ICS) [16]. We previously showed that the increased cyclical stretch of macrophages, which macrophages would experience in the intestinal wall during the development of edema or ileus, increases the secretion of inflammatory mediators such as CXCL1 and IL-1β [11]. Thus, the conditioned media from THP1 cells subjected to ICS contain inflammatory mediators, whereas the media from THP1 cells subjected to CCS do not contain inflammatory mediators. The effects of ZD7288 on intestinal contractile activity were measured after pretreating intestinal sections with the CCS or ICS “conditioned” media from THP1 cells. Spontaneous intestinal contractile activity was modestly but significantly decreased after treatment with ECS media compared with CCS treatment (Figure 3A). Inhibition of HCN with ZD7288 significantly suppressed spontaneous contractile activity, regardless of the media treatment (Figure 3B). Likewise, the pretreatment of intestinal sections from THP-1 media subjected to CCS or ICS did not significantly affect agonist-induced contractile activity (Figure 3C). Overall, the effects of HCN inhibition were similar in the presence or absence of inflammatory mediators secreted by macrophages.

### 2.4. Impact of Stretch on the Effects of HCN Inhibition

**Figure 4:**
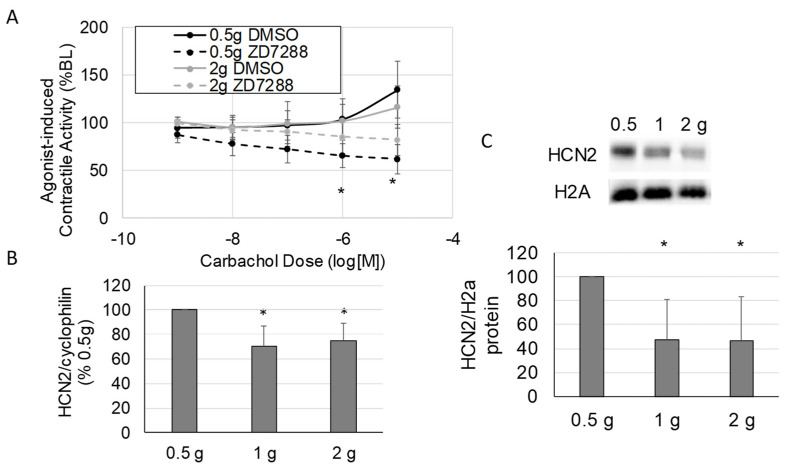
The impact of stretch on HCN2 inhibition of intestinal contractile activity. (**A**) Changes in the inhibitory effects of HCN inhibition on agonist-induced contractile activity in response to stretch. (**B**,**C**) The effects of increased stretch on smooth muscle tissue HCN2 mRNA (**B**) and protein (**C**) levels after applying 0.5 g (normal load), 1 g, and 2 g loads to intestinal tissue in the organ bath system. (VEH is DMSO only; Panel (**A**), *n* = 5 per group; Panels (**B**,**C**), *n* = 4–8 per group; Panel (**A**): *, *p* < 0.05 vs. DMSO at the same carbachol dose; Panels (**B**,**C**): *, *p* < 0.05 vs. 0.5 g load).

When ileus develops, the small intestine becomes highly distended and the intestinal wall is stretched. In addition, inflammatory conditions that provoke ileus also induce intestinal edema, resulting in distension of the intestinal wall. To examine the effects of HCN2 inhibition in the context of increased intestinal wall stretch, we inhibited HCN (50 μM ZD7288) in the presence of increased stretch or normal stretch (2 g preload vs. 0.5 g preload) and assessed the response to carbachol (Figure 4). As shown in Figure 4A, HCN inhibition significantly decreased agonist-induced contractile activity in tissues subjected to normal stretch (0.5 g preload + DMSO vs. 0.5 g preload + ZD7288, *p* < 0.05) but did not significantly decrease agonist-induced contractile activity when tissues were subjected to increased stretch (2 g preload + DMSO vs. 2 g preload + ZD7288). Thus, the effects of HCN inhibition were attenuated when tissues were stretched (2 g).

Based on a previous microarray study showing that HCN2 expression was decreased in intestinal smooth muscle when ileus was induced [19], we speculated that the attenuated effects of HCN inhibition after increased stretch were due to the down-regulation of HCN2. Thus, we measured HCN2 expression levels in stretched (2 h, 1 or 2 g preload) and unstretched (0.5 g preload) intestinal smooth muscle tissue. The mRNA levels for HCN2 decreased significantly in response to increased stretch (0.5 g preload vs. 1 or 2 g preload, *p* < 0.05 for both) compared to normal stretch (0.5 g preload) (Figure 4B). As shown in Figure 4C, both 1 and 2 g preloads also significantly decreased HCN2 protein levels in intestinal smooth muscle tissue (*p* < 0.05 vs. 0.5 g). Thus, HCN2 decreased in response to increased stretch, and at least part of the decrease in HCN2 protein levels in intestinal smooth muscle tissue in response to increased stretch was due to regulation at the transcriptional level.

### 2.5. Cell-Specific Mechanotransduction Effects on HCN2 Expression

**Figure 5:**
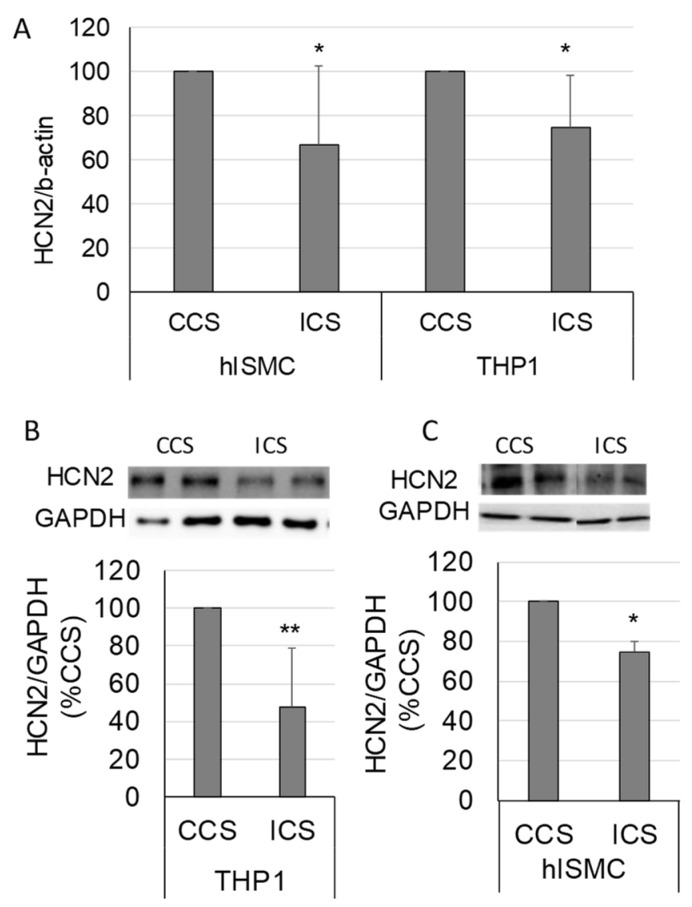
Changes in HCN2 mRNA and protein levels in response to increased cyclical stretch in primary human intestinal smooth muscle cells (hISMC) and THP1 cells (macrophage-like cell line). (**A**) HCN2 mRNA levels normalized to β-actin in hISMC and THP-1 cells after 4 h of controlled cyclical stretch (CCS) or increased cyclical stretch (ICS). (**B**,**C**) HCN2 protein levels in THP-1 (**B**) and hISMC (**C**) after 4 h of CCS or ICS normalized to GAPDH. (*n* = 7 and 5 per group in hISMC and THP-1 cells, respectively, in Panel (**A**); *n* = 6 per group in Panel (**B**); *n* = 3 per group in Panel (**C**); *, *p* < 0.05 vs. VEH; **, *p* < 0.005 vs. VEH).

Intestinal smooth muscle tissue comprises multiple cell types. Thus, we wanted to verify that the decreased HCN2 expression in tissues occurred in smooth muscle cells. In addition, macrophages play a significant role in the development of ileus [14]; thus, we also determined if HCN2 expression changed in macrophages in response to stretch. HCN2 mRNA levels decreased in both primary human intestinal smooth muscle cells and differentiated THP1 cells in response to increased cyclical stretch (CCS vs. ICS, *p* < 0.05 for both THP1 and hISMC) (Figure 5A). As shown in Figure 5B,C, HCN2 protein levels also decreased in both hISMC and THP1 cells in response to increased cyclical stretch (CCS vs. ICS, *p* < 0.05 for both THP1 and hISMC).

## 3. Materials and Methods

### 3.1. Materials

The HCN inhibitors ZD7288 and zatebradine were purchased from BioTechne (Minneapolis, MN, USA), and CXCL1 was purchased from R&D Systems (Abington, UK). Carbachol was purchased from VWR International (Debrecen, Hungary). Tetrodotoxin (TTX) was purchased from Tocris Bioscience (Bristol, UK).

### 3.2. Contractile Activity

All experiments were performed in FVB/Ant strain mice that were 3–6 months old. All animal experiments were approved by the Animal Welfare Committee of the University of Debrecen (1 April 2018). Six to eight mice per group were used for each experiment. Small intestinal strips (approximately 10 mm in length), four from each animal, were mounted in 20 mL organ baths (MDE, Walldorf, Germany) filled with Krebs-Ringer solution (in mM: 103 NaCl, 4.7 KCl, 2.5 CaCl_2_, 25 NaHCO_3_, 1.1 NaH_2_PO_4_, and 15 glucose). The solution was heated to 37 °C and gassed with 5% CO_2_–95% O_2_. Isometric force was monitored by an external force displacement transducer (Grass FT.03; Grass Instrument, Quincy, MA, USA) connected to a PowerLab (AD Instruments, Colorado Springs, CO, USA). Contractile activity was recorded continuously during the treatment protocols. The AD instruments system was calibrated using a small weight; thus, force (change in length) was measured in grams. After mounting, each strip was adjusted to a 0.5 g load, allowed to warm for 10 min, then readjusted to 0.5 g, and allowed to equilibrate for another 20 min. After equilibration, 10 min of basal contractile activity data were recorded (baseline). Both spontaneous (unstimulated) and agonist-induced (carbachol) contractile activities were measured. Spontaneous contractile activity developed within 2–3 min of mounting the tissue. Total contractile activity was calculated as the integral (area under the curve) in the AD Instruments software, and data over 4 min were averaged for each measurement. All measurements were normalized to baseline for each tissue section.

Treatments were added directly to the bath. A spontaneous contractile activity response to drug treatment was measured as a percent of the baseline for each tissue section. Agonist-induced contractile activity was measured as a percent of the last treatment before carbachol treatment. The effects of the HCN inhibition on spontaneous contractile activity (Figure 1) were measured by adding 50 μM ZD7288 to the organ bath containing ileum intestinal segments and recording 10 min of data. The dose–response to the HCN2 inhibitor (ZD7288) was measured at different doses of ZD7288 (0, 0.5, 5, and 50 μM) (Figure 1D). The calcium sensitivity curves were generated by pretreating intestinal tissue with 50 μM ZD7288 for 5 min in low calcium Krebs buffer (0.0025 mM Ca^2+^), followed by increasing concentrations of calcium (0.025 mM, 0.25 mM, and 2.5 mM Ca^2+^) added to the organ bath (Figure 1E).

For agonist-induced contractile activity, carbachol dose–response curves were generated by adding escalating doses of carbachol (in M: 10^−9,^ 10^−8^, 10^−7^, 10^−6^, 10^−5^, and 10^−4^) in 5 min intervals and the maximum agonist-induced contractile activity was determined (Figure 2A). The effects of zatebradine on agonist-induced contractile activity were determined in a similar manner, i.e., treatment with 50 μM zatebradine followed by the generation of carbachol dose–response curves (Figure 2C). To determine if HCN affected contractility via the smooth muscle or enteric nervous system, tissue was pretreated with 1 μM tetrodotoxin (TTX) for 5 min before treating with 50 μM ZD7288 for 5 min (Figure 2D).

To measure the influence of stretch on the effects of HCN inhibition, tissue was equilibrated at a 0.5 g load. After equilibration, tissue was left at 0.5 g preload or adjusted to a 1 g or 2 g preload. After 10 min, the tissue was readjusted to a 0.5 g preload, and a carbachol dose–response curve was generated. To measure the influence of inflammation on the HCN inhibition effects, tissue was pretreated with 10% conditioned media from macrophages (THP1 cells) before generating the carbachol dose–response curves. The collection of macrophage-conditioned media is described below.

### 3.3. Cells

Human intestinal smooth muscle cells (hISMC) were purchased (Provitro AG, Berlin, Germany). The hISMC were cultured in smooth muscle growth media (Sigma-Aldrich, Budapest, Hungary). Human acute monocytic cells (THP1) were ordered from Sigma Aldrich, Budapest, Hungary) and grown in RPMI 1640 media (Sigma-Aldrich, Budapest, Hungary). THP1 cells were activated with phorbol-12-myristate-13 acetate (100 ng/mL) 24 h before use.

### 3.4. Cell Stretching Protocols

The hISMC and THP1 cells were subjected to 4 h of control cyclical stretch (CCS), mimicking basal physiological conditions, or edema cyclical stretch (ECS), mimicking the mechanical stretch induced by edema development in the gut wall, as previously described [16,20]. The 4 h cyclical stretch protocols were designed using the spontaneous contraction amplitude, frequency, and tension measured in vivo in previous experiments [15,16,20]. Briefly, cells were seeded onto Bioflex 6-well plates (collagen I-coated, Dunn Labortechnik Gmbh, Asbach, Germany), which were pretreated for 24 h with 0.1% M poly-L-lysine, at a density that would enable them to reach 70–90% confluency on the following day. On the day of the experiment, the cells were subjected to CCS or ECS. The CCS protocol consisted of cyclical stretch at 0.4 Hz (sinusoidal curves) at a minimum of 1% and a maximum of 3% elongation for 4 h. The ECS protocol consisted of cyclical stretching (0.4 Hz, sinusoidal wave) with increasing elongation; cycling between 1–3% and increasing to 18–20% over 30 min. Cyclical stretching was maintained at 18–20% for the remaining 3.5 h.

### 3.5. Macrophage-Conditioned Media

For macrophage-conditioned media, THP1 cells were seeded onto Flexcell plates and activated with phorbol-12-myristate-13-acetate (PMA, 100 ng/mL). After 24 h, cells were subjected to CCS or ICS, as described above. After stretching, media were collected and stored at −80 °C until use. When treating tissue with conditioned media, 10% of the buffer in the organ bath chamber was replaced with conditioned media.

### 3.6. Western Blot

In tissue, the mucosa was removed before preparing protein lysates. Proteins were separated by sodium-dodecyl sulfate polyacrylamide gel (7.5%) electrophoresis and probed with antibodies, as previously described [16]. The following antibodies were used: anti-HCN2 (1:1000, SAB5200025; Sigma Aldrich. Kft., Budapest, Hungary), anti-H2A (1:1000, 3636S; Cell Signaling Technology, Danvers, MA, USA), and anti-IgG-HRP (1:5000, Santa Cruz Biotechnology, Heidelberg, Germany).

### 3.7. Reverse Transcriptase Quantitative PCR

RNA was isolated from frozen tissue or cells using Tri Reagent (Sigma-Aldrich, Budapest, Hungary) following the manufacturer’s directions. The RNA samples were DNase treated immediately after RNA isolation to remove genomic DNA contamination. Reverse transcription was performed on 1 μL RNA in triplicate at 50 °C for 30 min using 1× RT buffer, 400 nM specific reverse primer, 500 μM dNTPs, and Superscript II (LTI, Bethesda, MD). After 5 min at 72 °C, the RT reaction (5 μL) was added to 20 μL of PCR mix containing 1× PCR buffer, 400 nM primers, 2 mM MgCl_2_, Taq Polymerase (0.02 U/μL), and 100 nM fluorogenic probe. Amplification and quantitation based on real-time monitoring of the amplification were carried out with 40 cycles of 94 °C for 20 s and 60 °C for 30 s, following a 1 min denaturation step at 95 °C. The values of transcripts in unknown samples were obtained by interpolating their Ct (PCR cycles to threshold) values on a standard curve derived from known amounts of cognate, specific amplicons. Transcript levels were normalized to the level of β-actin or cyclophilin RNA. All determinations were performed in triplicate and with no template and –RT controls. The sequences of all primers and TaqMan probes used for amplification reaction assays are summarized in Table 1.

### 3.8. Statistical Analyses

Data are presented as means ± standard deviations. Two groups were compared with *t*-tests and multiple groups were compared with ANOVA with Fisher’s LSD post hoc test. The carbachol dose–response curves were compared using a two-way ANOVA with carbachol dose and treatment as the independent variables. Data were analyzed using the Excel and Statistica software programs. Contractile activity was normalized to baseline for each tissue section. PCR and Western blot data were normalized to the corresponding control in each individual experiment.

## 4. Discussion

Although mechanotransduction clearly plays a role in both the normal physiology and pathology of the gastrointestinal tract [16,34], the role of mechanotransduction in gastrointestinal motility disorders, including ileus, is largely unexplored. To investigate the role of mechanotransduction in the development of ileus, we searched the data from an earlier microarray study for mechanosensitive ion channels that are altered after the induction of ileus. In a rodent model of ileus, HCN2 was significantly down-regulated in intestinal smooth muscle compared to controls [19]. Thus, the objective of this study was to determine the effects and mechanism of HCN2 down-regulation on intestinal motility. Our results show that the inhibition of HCN channels significantly decreases intestinal contractile activity by decreasing intestinal tone but not contraction amplitude (Figure 1A–C). The down-regulation of both HCN2 mRNA and protein levels was induced by increased mechanical stretch of the intestinal wall (Figure 4B,C). Overall, our results suggest that decreased HCN2 expression induced by mechanical signals may contribute to the development of ileus.

The role of HCN2 in intestinal motility is unknown. HCN2 expression was detected in enterochromaffin cells and may play a role in serotonin release in enterochromaffin cells, which may affect intestinal motility [35]. HCN2 is also highly expressed in myenteric cholinergic neurons and expressed at higher levels in the ileum compared to the duodenum or jejunum [33]. Yang et al. showed that anti-Kit-positive cells were not positive for HCN2 staining [33], indicating that HCN2 was not expressed in interstitial cells of Cajal (ICCs). However, Guo et al. detected HCN2 in isolated ICCs [26] and suggested that HCN2 plays a pacemaker role. The frequency of contraction tended to decrease in our study; however, the difference was not significant (*p* = 0.14). Furthermore, intestinal tone, which should not be affected by contraction frequency, was significantly decreased in the small intestine in response to HCN inhibition (Figure 1C). Thus, effects on pacemaker activity in the small intestine in response to HCN inhibition cannot entirely explain the suppression of intestinal contractile activity in the small intestine in response to HCN inhibition. Tetrodotoxin did not block the effects of HCN inhibition of either spontaneous contractile activity or agonist-induced contractile activity (Figure 2D); however, HCN inhibition suppressed calcium sensitivity in the small intestine. Overall, our results indicate that at least part of the effects of HCN inhibition on intestinal motility is due to effects on intestinal smooth muscle cells.

To the best of our knowledge, we are the first to demonstrate HCN2 expression in isolated intestinal smooth muscle cells. We detected HCN2 by both RT-qPCR and Western blotting in primary human intestinal smooth muscle cells. Inhibition of HCN2 caused decreased spontaneous and agonist-induced intestinal contractile activity (Figure 1 and Figure 2). In agreement with our results, Fisher et al. showed that the loss of HCN2 protein leads to delayed gastrointestinal transit [31]. Interestingly, HCN expression is altered in several diseases associated with gastrointestinal motility disorders, including Hirschsprung’s disease and Parkinson’s disease [36,37]. Overall, our data combined with the Fisher study support the fact that HCN2 plays a role in intestinal motility.

Surprisingly, our results indicate that HCN2 affects intestinal tone but not contraction amplitude and does not significantly affect the frequency of spontaneous contractile activity (Figure 1A–C). There is some evidence that sustained contractions, i.e., intestinal tone, are regulated differently than phasic contractions of the intestine, i.e., contraction amplitude in this study. Sustained, but not transient, contractions in the ileum can be regulated by Rho-associated kinase, suggesting that slow-cycling crossbridges are regulated differently than fast-cycling crossbridges [38,39]. Rho-associated kinase also plays a significant role in agonist-induced contractile activity [40], which was profoundly suppressed by HCN2 inhibition in our study (Figure 2). However, the role of HCN2 in sustained contractions is unknown. Thus far, HCN2 involvement in pacemaker activity has been demonstrated; thus, the mechanism by which HCN2 can affect sustained contractile activity is unknown. HCN2 inhibition affected calcium sensitivity in the ileum and duodenum (Figure 1E). Yu et al. showed that calcium ions can enter myocytes through HCN channels [41]. Although the relative proportion of calcium influx through HCN channels was low, the down-regulation of HCN2 could reduce calcium influx and, therefore, affect intestinal tone. HCN2 has also been shown to interact with L-type calcium channels that are at least partially responsible for agonist-induced calcium influx in intestinal smooth muscle cells [42,43]. HCN2 channels also modulate subthreshold membrane potentials in response to cyclic nucleotides [44]. Changes in HCN2 may also affect other pathways. For example, the inhibition of HCN2 decreased NF-κB p65 activation and the release of inflammatory mediators in ipsilateral spinal dorsal horns [45]; changes in these inflammatory pathways may affect intestinal motility. Thus, the down-regulation of HCN2 may affect contractile activity through direct effects on calcium influx, interactions with other ion channels, or effects on other signaling pathways.

Inflammation plays a substantial role in the development of ileus. Resident macrophages in the intestinal wall are activated, leading to the recruitment of neutrophils and the release of inflammatory mediators [10,12,14]. Furthermore, multiple inflammatory mediators affect intestinal motility [46]. Thus, the impact of inflammation on HCN effects was examined. We previously showed that the increased cyclical stretch of macrophages, which occurs during the development of intestinal edema or ileus, increases the secretion of inflammatory mediators [11]. Thus, we tested the effects of conditioned media from macrophages after control and increased cyclical stretch on contractile activity after HCN inhibition. The treatment of intestinal sections with conditioned media from macrophages subjected to increased cyclical stretch inhibited contractile activity but did not significantly affect the inhibition of intestinal contractile activity in the presence of HCN inhibitors (Figure 3). However, these experiments were inconclusive and the effects of individual inflammatory mediators on HCN expression and the inhibition of contractile activity should be further explored.

HCN inhibition of agonist-induced contractile activity was partially attenuated when intestinal sections were subjected to increased stretch before treatment with the HCN inhibitor (Figure 4A). Thus, we checked if the expression of HCN2 changed in the intestinal tissue. We found that HCN2 was significantly decreased when tissue was subjected to increased mechanical stretch (Figure 4B,C). These findings were confirmed in human intestinal smooth muscle cells (Figure 5) and are consistent with the previously mentioned microarray data [19]. HCN2 mRNA levels in smooth muscle tissue did not decrease to the same extent as the protein levels, suggesting that protein degradation may also contribute to the decreased HCN2 protein levels. As macrophages play a significant role in the development of ileus, we also examined the expression of HCN2 in macrophages (Figure 5A,C). To our knowledge, the expression or function of HCN channels in macrophages has not been demonstrated. However, HCN channels may play a role in microglia, a specialized macrophage in the central nervous system [47,48].

The mechanotransductive mechanism affecting HCN2 expression is unclear. To our knowledge, this is the first study to demonstrate the down-regulation of HCN2 in response to mechanical stretch (Figure 4 and Figure 5). The regulation of HCN2 expression has not been widely explored. TRIP8b antagonizes the activity of HCN1–4 and also regulates HCN trafficking and expression in a complicated manner [49]. SUMOylation increases the expression of HCN2 on the membrane surface of human embryonic kidney cells [50], and SUMOylation can be regulated by mechanotransductive forces [51,52]. There is some evidence that HCN2 interacts with the cytoskeleton [53], and interactions with the cytoskeleton could mediate stretch-induced changes in expression. Overall, little is known about the regulation of HCN2 in general; thus, the mechanism of the mechanoregulation of HCN2 is unknown. HCN2 expression in neurons is regulated by inflammation in some models [54,55]; however, inflammation did not appear to modulate the effects of HCN2 inhibition in our model (Figure 3).

This study has several limitations. First, although we showed HCN2 mRNA and protein expression in human intestinal smooth muscle cells, we did not show the immunohistochemical localization of HCN2 to intestinal smooth muscle cells. In addition, we did not measure changes in HCN2 in enteric neurons. HCN2 is expressed in myenteric cholinergic neurons, and changes in HCN2 expression in the enteric neurons may have occurred in response to tissue stretch. Both HCN inhibitors used in this study are not specific for HCN2 and may have also inhibited HCN1. Unfortunately, there are no specific HCN2 inhibitors available at this time.

In summary, the inhibition of HCN significantly decreased intestinal motility by decreasing intestinal tone but not contraction amplitude. HCN inhibition also profoundly inhibits agonist-induced intestinal contractile activity. HCN2 mRNA and protein levels were down-regulated by increased mechanical stretch of the intestinal wall. Overall, our results suggest that decreased HCN2 expression induced by mechanical signals may contribute to the development of ileus.

## Figures and Tables

**Table 1:** Primers, probes, and sAmplicons for RT-qPCR assays.

Primer/Probe/sAmp	Primer Sequence
hHCN2+	ATCCACCCGTACAGCGACTT
hHCN2−	GATGAGGTTTCCCACCATGAA
h/mHCN2 probe	AGGTTCTACTGGGACTTCACCATGCTGCT
hHCN2 sAmp	CATCCACCCGTACAGCGACTTCAGGTTCTACTGGGACTTCACCATGCTGCTGTTCATGGTGGGAAACCTCATCA
mHCN2+	CATCCACCCCTACAGCGACTT
mHCN2−	CCCACGGGAATGATAATGAGA
mHCN2 sAmp	TCATCCACCCCTACAGCGACTTCAGGTTCTACTGGGACTTCACCATGCTGCTGTTCATGGTGGGAAATCTCATTATCATTCCCGTGGGC
mCyclophilin+	CGA TGA CGA GCC CTT GG
mCyclophilin−	TCT GCT GTC TTT GGA ACT TTG TC
mCyclophilin probe	CGC GTC TCC TTC GAG CTG TTT GCA
mCyclophilin sAmp	CGATGACGAGCCCTTGGGCCGCGTCTCCTTCGAGCTGTTTGCAGACAAAGTTCCAAAGACAGCAGA
hB-actin+	CCC TGG CAC CCA GCA C
hB-actin−	GCC GAT CCA CAC GGA GTA C
hB-actin probe	ATC AAG ATC ATT GCT CCT CCT GAG CGC
hB-actin sAmp	TGC CCT GGC ACC CAG CAC AAT GAA GAT CAA GAT CAT TGC TCC TCC TGA GCG CAA GTA CTC CGT GTG GAT CGG CGG

## Data Availability

The primary data are available upon reasonable request.
